# Expression and Prognostic Significance of Wnt7a in Human Endometrial Carcinoma

**DOI:** 10.1155/2012/134962

**Published:** 2012-12-06

**Authors:** Chunjie Peng, Xiaolei Zhang, Yujie Wang, Lijuan Li, Qiuyan Wang, Jianhua Zheng

**Affiliations:** ^1^Department of Obstetrics and Gynecology, Harbin Red Cross Hospital, Harbin 150076, China; ^2^Department of Obstetrics and Gynecology, The First Affiliated Hospital of Harbin Medical University, no. 23 Youzheng Street, Nangang Block, Harbin 150010, China; ^3^Department of Obstetrics and Gynecology, The Fifth Hospital of Daqing, Heilongjiang, China

## Abstract

*Introduction*. Wnt7a is a secreted glycoprotein that regulates normal cellular proliferation and differentiation as well as tumorigenesis and progression. However, less is understood about the role of Wnt7a in human endometrial carcinoma. The aim of this study is to investigate the expression and prognostic significance of Wnt7a in endometrial carcinoma. *Methods*. Wnt7a expression was immunohistochemically examined in 35 normal endometrium, 33 hyperplastic endometrium and 70 endometrial carcinomas. *Results*. Wnt7a expression was lower in endometrial carcinomas compared with that in normal and hyperplastic endometrium (*P* < 0.001). Wnt7a was inversely correlated with FIGO stage (*P* = 0.001), grade (*P* = 0.001), lymph node metastasis (*P* = 0.002), depth of myometrial invasion (*P* = 0.006), lymph vascular space involvement (*P* = 0.001) and peritoneal cytology (*P* = 0.013). There was a negative correlation between estrogen receptor (ER) and Wnt7a (*r* = −0.281, *P* = 0.019), and a positive correlation between progestogen receptor (PR) and Wnt7a (*r* = 0.249, *P* = 0.037). Patients with lost or reduced Wnt7a expression had poorer progression-free survival (PFS) and overall survival (OS) (*P* = 0.005 and *P* = 0.042, resp.) on univariate analysis. But on multivariate analysis, Wnt7a expression was not an independent prognostic factor for PFS or OS. *Conclusions*. Our results indicate that Wnt7a expression may serve as an important prognostic marker.

## 1. Introduction

Endometrial carcinoma is the most common malignancy of the female genital tract worldwide, and it is the seventh leading cause of death among women suffering from cancer [1, 2]. Frequently, this malignancy is diagnosed at an early stage and has a better prognosis than other cancers. However, advanced and recurrent cases carry a poor prognosis, because of responding poorly to the conventional treatments such as cytotoxic chemotherapy, radiotherapy, or hormonal therapy. Although the stage, grade, histological subtype, and tumor size correlated with the outcome, identification of specific molecular markers could contribute more to predict the prognosis of endometrial carcinomas.

Wnt proteins comprise a large family of secreted, highly conserved glycoproteins that exhibit pivotal role in early mammalian development. They are associated with important cellular process such as proliferation, differentiation, cell fate determination, apoptosis, and carcinogenesis [3–5]. A Wnt protein as a ligand binds to a specific receptor located on the cell surface, activating the canonical or noncanonical pathway: Wnt/*β*-catenin canonical pathway. Without the Wnt signaling, activated glycogen synthase kinase 3 (GSK3) phosphorylates adenomatous polyposis coli (APC) and Axin and increases their activities to degrade *β*-catenin effectively. Wnts interacting with the Frizzled (Fzd) receptor and low-density lipoprotein receptor-related protein 5 and 6 (LRP5/6) receptor can inactivate GSK3 by phosphorylation. When GSK3 is inactivated by Wnt signaling, cytoplasmic *β*-catenin accumulates and translocates to the nucleus, where it regulates target gene transcription depending on transcription factors, T cell factor (TCF)/lymphoid enhancer factor (LEF) family [6]. The noncanonical pathway is transduced through Fzd independent on LRP5/6 or *β*-catenin. Wnt7a mainly activates the canonical pathway; however, Wnt7a signaling mediated by Fzd10 induced a noncanonical c-Jun NH_2_-terminal kinase responsive pathway [7].

Wnt7a plays a pivotal role in development. Mice lacking Wnt7a had malformed female reproductive tracts [8], and mice treated with carcinogen diethylstilbestrol (DES) exhibited lower Wnt7a expression level and some abnormalities in their uteri. They also showed signs of cervical and/or vaginal adenocarcinomas by six months after birth, thus indicating involvement of Wnt7a in carcinogenesis [9]. In non-small-cell lung cancer (NSCLC) cells, the restoration of Wnt7a and Fzd9 signaling inhibited cell proliferation, promoted cell differentiation, and reversed the transformed phenotype, suggesting that Wnt7a behaves as a tumor suppressor gene [10]. In accordance with the suppressing effects in NSCLC, Wnt7a was also found to be hypermethylated at high frequency in pancreatic carcinoma [11]. However, in contrast to the studies above, Wnt7a was overexpressed in the cell lines of colorectal carcinoma, pancreatic carcinoma, gastric carcinoma, and breast carcinoma [12]. In addition, Wnt7a expression was exclusively high in the malignant ovarian carcinomas and involved in increased migration and invasive capacity in an ovarian cancer cell line OVCAR3, acting as an oncogenic gene [13]. These results indicate that there may be a contrary effect of Wnt7a in different carcinomas.

Wnt7a has also been detected in endometrial carcinoma cell lines and endometrial tumors [14]. In the endometrium, Wnt7a had a function in cell-cell communication and was responsive to different levels of sex steroid hormones [15]. The estrogen receptor (ER) antagonist could reverse the reduction of Wnt7a by estrogens in endometrial carcinoma cell line, thus indicating that the downregulation of Wnt7a was partly mediated by the ER [16]. Endometrial carcinoma is hormone-dependent malignant disease, and ER and progestogen receptor (PR) expression correlate with tumorigenesis and clinicopathological features. Previous studies on Wnt7a expression were mainly at the mRNA level, and few were at the protein level. In the present study, we investigated Wnt7a protein expression in the normal endometrium, hyperplastic endometrium, and endometrioid endometrial carcinomas by immunohistochemistry. Furthermore, we analyzed the association of Wnt7a expression with clinicopathological characteristics and outcome of patients with endometrial carcinomas and evaluated the correlation between Wnt7a expression and the hormone receptor status.

## 2. Patients and Methods

### 2.1. Patients and Tissue Specimens

70 endometrioid endometrial adenocarcinomas, 33 endometrial hyperplasia (16 simple endometrial hyperplasia, 17 complex endometrial hyperplasia), and 35 normal endometrium (12 proliferative endometrium, 13 secretory endometrium, 10 atrophic endometrium) between January 2000 and May 2004 were selected from the Tumor Hospital of Harbin Medical University after informed consent. All patients with endometrial carcinoma were surgically treated, and before surgery without any chemotherapeutic or radiotherapeutic treatment. Differentiation grade was assigned according to the World Health Organization (WHO) Criteria and was further reviewed by two experienced pathologists: 30 were grade 1, 25 were grade 2, and 15 were grade 3. Surgical staging was reviewed based on the International Federation of Gynecology and Obstetrics (FIGO) System: 30 cases were allocated to stage I, 9 to stage II, 26 to stage III, and 5 to stage IV. Progression-free survival (PFS) was defined as the time interval between the date of surgery and the date of identification of progressive disease. Overall survival (OS) was defined as the time interval between the date of surgery and the date of death.

### 2.2. Immunohistochemistry

Formalin-fixed, paraffin-embedded samples were cut into sections of 4 *μ*m thickness, then the sections were deparaffinized with xylene and rehydrated in serial dilutions of ethanol. Endogenous peroxidase activity was quenched by 3% hydrogen peroxide at room temperature for 10 min. After that, the sections were heated in microwave for 20 min in 10 mM trisodium citrate buffer (PH 7.0) to retrieve antigens and then blocked with normal goat serum for 30 min. The sections were incubated overnight at 4°C with the primary antibodies for Wnt7a (goat anti-Wnt7a, diluted 1 : 80, AF3008; R&D, Minneapolis, MN, USA), ER (rabbit anti-ER, diluted 1 : 300, sc-543; Santa Cruz Biotechnology, Santa Cruz, CA, USA), and PR (rabbit anti-PR, diluted 1 : 200, sc-538; Santa Cruz Biotechnology, Santa Cruz, CA, USA). After washing, the signal was amplified by incubating the sections with secondary antibodies for 45 minutes at 37°C; anti-goat IgG-polymer horseradish peroxidase (HRP; Polink-2 Plus HRP Goat DAB kit, D43-6; GBI, Mukilteo, WA, USA); anti-rabbit IgG-polymer horseradish peroxidase (HRP; Polink-2 Plus HRP Rabbit DAB kit, D39-6; GBI, Mukilteo, WA, USA). Finally, the reaction was revealed with a 3,3′-diaminobenzidine solution. Subsequently, the sections were counterstained with hematoxylin. Pancreatic carcinomas were used as a positive control for Wnt7a and breast carcinomas for ER/PR, respectively. The primary antibodies were replaced with phosphate-buffered saline (PBS) as a negative control.

### 2.3. Staining Evaluation

The Wnt7a expression level was classified based on the total combined scores of the percent positive staining tumor cells together with the staining intensity. The percentage of positive tumor cells was graded and scored as follows: 0 (none), 1 (1–25%), 2 (26–50%), 3 (51–75%), and 4 (76–100%). The staining intensity was graded and scored as 0 (no staining), 1 (very weak), 2 (weak), 3 (moderate), and 4 (strong). The final score (0–8) was calculated by the sum of the positive score and the staining intensity score. Scores from 0 to 2 were considered as negative, and scores ≥3 were considered as positive. As for evaluation of ER and PR immunoreactivity, tumors with positive ER or PR nuclear staining in >10% of tumor cells were defined as ER or PR positive, respectively. All the histological slides were examined by two observers who were unaware of the clinical data or the disease outcome.

### 2.4. Statistical Analyses

The Chi-square test or Fisher's exact test was used to examine the association between the expression of Wnt7a and these clinicopathological features such as age, FIGO stage, grade, lymph node metastasis, depth of myometrial invasion, peritoneal cytology, and lymph vascular space (LVS) involvement. Survival curves were plotted using the Kaplan-Meier method and differences between survival curves were tested using the log-rank test. For multivariate analysis, Cox proportional-hazard model was performed. Spearman's rank correlation test was used to analyze the correlation between the Wnt7a expression and ER/PR expression in endometrial cancer. These analyses were performed using SPSS software Version 13.0. *P* < 0.05 was considered statistically significant.

## 3. Results

### 3.1. Characteristics of the Patients

The demographic features of the patients are listed in Table 1. All tumors were of endometrioid histology. 55.7% of patients had stage I or II disease, and 57.1% of tumors were poorly differentiated. The median follow-up time for patients in this study was 65 months. 

### 3.2. Wnt7a Expression in Human Endometrial Tissues

**Figure 1:**
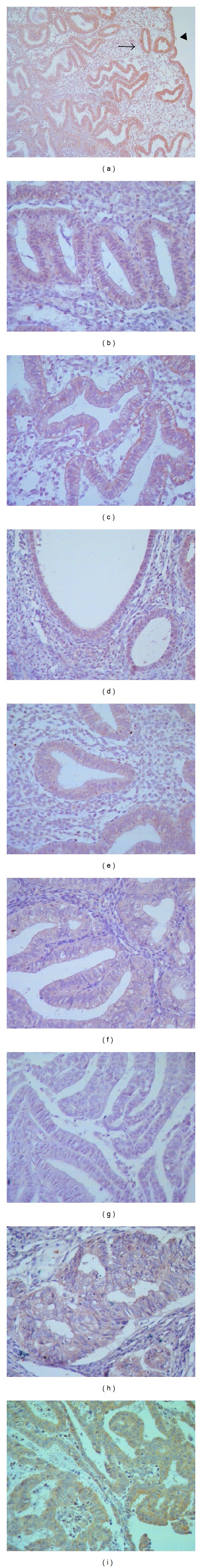
Wnt7a protein expression in endometrial tissues. (a) Wnt7a staining in the glandular epithelial cells (arrow) and in the luminal epithelial cells (triangle) (×100); (b) proliferative endometrium (×400); (c) secretory endometrium (×400); (d) atrophic endometrium (×400); (e) simple hyperplasia (×400); (f) complex hyperplasia (×400); (g) endometrial carcinoma with no Wnt7a staining (×400); (h) endometrial carcinoma with moderate Wnt7a staining (×400); (i) endometrial carcinoma with strong Wnt7a staining (×400).

**Figure 2:**
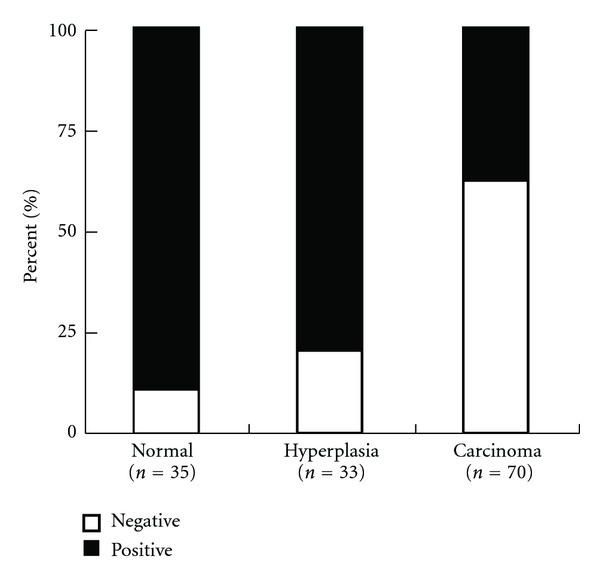
Overall level of Wnt7a expression in normal endometrium, hyperplastic endometrium and endometrial carcinomas according to the immunohistochemical result (endometrial carcinoma versus normal endometrium: *P* < 0.001; endometrium carcinoma versus hyperplastic endometrium: *P* < 0.001).

Wnt7a immunostaining was localized in the cytoplasm of the luminal and glandular epithelial cells (Figure 1(a)). As shown in Figures 1(b)–1(i), the immunoreactivity of Wnt7a was detected at variable levels. Wnt7a expression was lower in endometrial carcinomas (26/70, 37.1%) compared with that in normal endometrium (31/35, 88.6%) and hyperplastic endometrium (26/33, 78.8%) (*P* < 0.001 and *P* < 0.001, resp.) (Figure 2). However, the expression levels of Wnt7a were not significantly different between proliferative and secretory phase (*P* = 0.490) or between simple and complex endometrial hyperplasia (*P* = 0.737). Detailed expressions of Wnt7a in all endometrial tissues are shown in Table 2. Wnt7a expression was inversely correlated with FIGO stage (*P* = 0.001), grade (*P* = 0.001), lymph node metastasis (*P* = 0.002), depth of myometrial invasion (*P* = 0.006), LVS involvement (*P* = 0.001), and peritoneal cytology (*P* = 0.013) of endometrial carcinomas (Table 1). There was a negative correlation between ER and Wnt7a expression (*r* = −0.281, *P* = 0.019). A positive correlation between PR and Wnt7a was also detected (*r* = 0.249, *P* = 0.037) (Table 3). 

### 3.3. Correlation of Wnt7a Expression with Progression-Free Survival and Overall Survival

**Figure 3:**
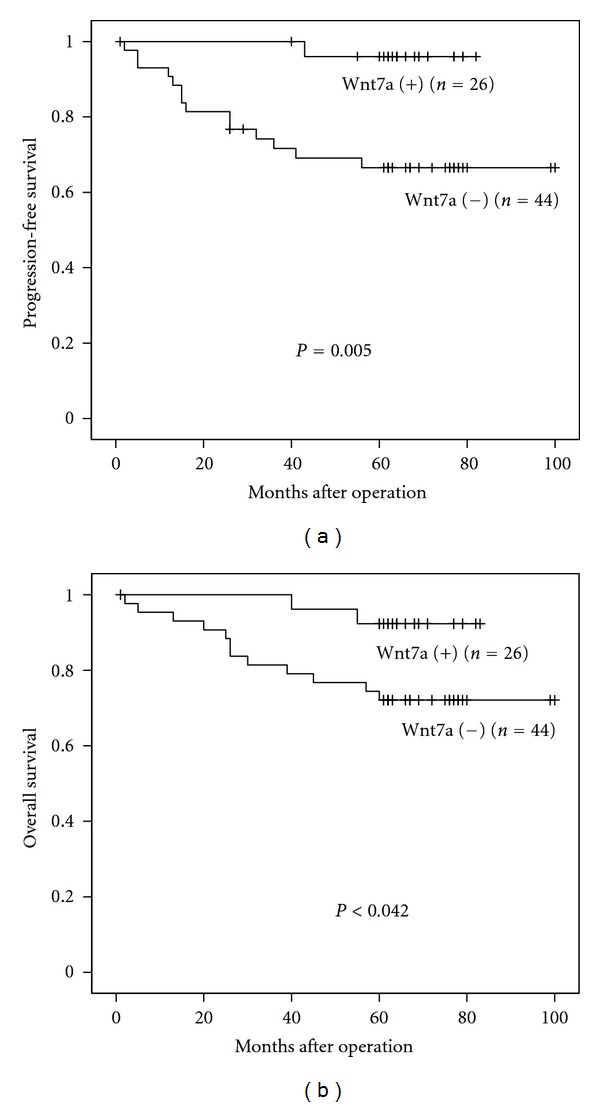
(a) Kaplan-Meier curves for PFS of endometrial carcinoma patients according to negative and positive expression of Wnt7a (log-rank analysis). (b) Kaplan-Meier curves for OS of endometrial carcinoma patients according to negative and positive expression of Wnt7a (log-rank analysis).

At the end of the follow-up period, 14 (20.0%) patients were dead of the disease. Only one of the patients was lost for followup. The 5-year OS rates of patients with negative and positive Wnt7a expression were 72.1% and 92.3%, respectively. And the 5-year PFS rates of patients with negative and positive Wnt7a expression were 66.5% and 96.0%, respectively. Both OS and PFS in patients negative for Wnt7a expression were significantly lower than those in patients who were positive for Wnt7a expression (*P* = 0.042 and *P* = 0.005, resp.) (Table 4). The Kaplan-Meier curves for PFS and OS of endometrial carcinoma patients according to negative and positive expression of Wnt7a were shown in Figure 3. The results of the univariate survival analyses of other variables are shown in Table 4. Multivariate analysis using the Cox proportional-hazard model indicated that peritoneal cytology, but not Wnt7a, was an independent prognostic factor (Table 5).

## 4. Discussion 

Previous studies have demonstrated the correlation between Wnt proteins' expression and prognosis in various human carcinomas [17, 18]. In the present study, we examined the status of Wnt7a expression in normal, hyperplastic, and malignant endometrium and analyzed its possible roles in the clinical outcome of patients with endometrial cancer. We could demonstrate for the first time that lost or reduced Wnt7a expression was correlated with disease progression and Wnt7a might be a useful prognostic factor in endometrial carcinoma.

In our immunohistochemical results, cytoplasmic Wnt7a was exhibited in both the luminal epithelial cells and the glandular epithelial cells, but not in the stromal cells. However, it has been reported that Wnt7a was expressed in the three kinds of cells above using in situ hybridization, real-time PCR of laser microdissected tissue, and immunofluorescence [19]. In contrast, Wnt7a mRNA expression exclusively in the luminal epithelium was documented in previous studies on mouse and human endometrium [4, 20]. First, the contradiction in the location may be due to different regulation mechanisms acted on synthesis and degradation of Wnt7a mRNA and protein. Second, different detection thresholds and methodologic approaches may also result in the discrepancy.

Absent or reduced Wnt7a expression was detected in the majority of endometrial carcinoma, but only in 21.2% of endometrial hyperplasia and 11.4% in normal endometrium. There was a significant difference of Wnt7a expression among the groups of normal, hyperplastic, and carcinomatous endometrium. In the normal endometrium, there was no correlation between Wnt7a expression and menstrual cycle, which is consistent with other studies on Wnt7a mRNA expression in human endometrium [14, 20]. However, a wide range of Wnt7a mRNA expression in endometrial carcinoma was not consistent with our present study [14]. This discrepancy is probably due to the different interpretations and methods used in the studies. Additionally, only four endometrial carcinoma samples selected in the earlier study were relatively small, compared with seventy samples in our present study.

We demonstrated that Wnt7a negative expression was inversely correlated with FIGO stage, grade, lymph node metastasis, depth of myometrial invasion, LVS involvement, and peritoneal cytology, indicating that Wnt7a functioned as a tumor-suppressing factor in endometrial carcinoma. Furthermore, lower Wnt7a expression was correlated with poor clinical outcome in univariate analysis, but it was not an independent prognostic factor in multivariate analysis. In fact, Wnt7a has recently been described to have a tumor suppressing effect in other studies. It was reported that progestogens upregulated Wnt7a gene expression in endometrial epithelial cells, suggesting that upregulation of Wnt7a may be associated with the tumor-suppressing effect of progestogens [21]. In non-small-cell lung carcinoma, the antitumorigenic effect of Wnt7a interacting with the specific receptor Fzd9 was mainly mediated through ERK-dependent activation of the nuclear receptor gene PPAR*γ* [10]. The Wnt7a gene was also found to be downregulated because of hypermethylation at high frequency in pancreatic carcinoma [11]. However, another study was in contrast to the opinion above concerning the tumor suppressing function of Wnt7a. Merritt MA et al. have observed that Wnt7a was overexpressed in invasive and low malignant potential ovarian tumor samples compared with benign and normal ovarian tissues. Meanwhile, in the OVCAR3 ovarian carcinoma cell line, stable overexpression of Wnt7a resulted in increased migration and invasive capacity [13]. Growing evidence from in vitro and in vivo studies suggests that Wnt7a may actually be able to do both, promote and suppress tumorigenesis. The functions of Wnt7a may therefore be dependent on the organ and tumor type examined. In addition, we speculate that the mechanism of Wnt7a is possibly based on the Fzd receptor availability, because at least 10 members of the Fzd family have been identified. Therefore, it is warranted to investigate the major Fzd receptor expression interacting with Wnt7a on the endometrial carcinoma cell surface.

Furthermore, the observed negative correlation between Wnt7a and ER status but positive correlation between Wnt7a and PR status in endometrial carcinoma indicated a link between Wnt7a and ovarian hormones. Studies have correlated sex hormones with Wnt7a expression in gynecologic diseases. In endometrial carcinoma Ishikawa cell line, estrogens decreased Wnt7a expression through its receptor ER [16]. Another study detected that progestogens upregulated Wnt7a gene in endometrial epithelial cells [21]. In, accordance with endometrial carcinoma, an inverse correlation was apparent between Wnt7a and ER*α* expression in human uterine leiomyoma [22]. These previous results indicate that decreased Wnt7a expression is associated with the development of sex-hormone-dependent endometrial carcinoma and leiomyoma.

In conclusion, we have shown that lost or reduced Wnt7a expression is significantly associated with poor progression-free and overall survival in patients with endometrial carcinoma. These results suggest for the first time that Wnt7a might play a role in tumor suppression in endometrial cancer. Although our results are derived from a limited number of patients, the present study provides important basis for future. Further studies on a larger cohort of patients and Wnt7a targeting molecules are warranted to validate the prognostic impact of Wnt7a expression on survival in endometrial cancer.

## Figures and Tables

**Table 1:** Relationship between the expression of Wnt7a and clinicopathological parameters of endometrial carcinoma.

Variable	Number	Wnt7a (%)		P*-value
		Negative	Positive
Total	70	44 (62.9)	26 (37.1)	
Age (years)				0.927
≤60	22	14 (63.6)	8 (36.4)	
>60	48	30 (62.5)	18 (37.5)	
FIGO stage				0.001
I+II	39	18 (46.2)	21 (53.8)	
III+IV	31	26 (83.9)	5 (16.1)	
Grade				0.001
G1	30	12 (40.0)	18 (60.0)	
G2 + G3	40	32 (80.0)	8 (20.0)	
Lymph node metastasis				0.002
Negative	49	25 (51.0)	24 (49.0)	
Positive	21	19 (90.5)	2 (9.5)	
Depth of myometrial invasion				0.006
≤1/2	31	14 (45.2)	17 (54.8)	
>1/2	39	30 (76.9)	9 (23.1)	
LVS involvement				0.001
Negative	44	21 (47.7)	23 (52.3)	
Positive	26	23 (88.5)	3 (11.5)	
Peritoneal cytology				0.013
Negative	53	29 (54.7)	24 (45.3)	
Positive	17	15 (88.2)	2 (11.8)	

FIGO: International Federation of Gynecology and Obstertics; LVS: lymph vascular space and *Chi-square test.

**Table 2:** The expressional profile of Wnt7a in endometrial carcinoma and its precursor lesions.

	Number	Wnt7a expression		P*-value
		Negative (%)	Positive (%)
Proliferative endometrium	12	2 (16.7)	10 (83.3)	0.004
Secretory endometrium	13	1 (7.7)	12 (92.3)	<0.001
Atrophic endometrium	10	1 (10.0)	9 (90.0)	0.002
Simple hyperplasia	16	3 (18.8)	13 (81.2)	0.002
Complex hyperplasia	17	4 (23.5)	13 (76.5)	0.004
Endometrial carcinoma	70	44 (62.9)	26 (37.1)	

Proliferative endometrium versus carcinoma: 0.004; Secretory endometrium versus carcinoma: <0.001; Atrophic endometrium versus carcinoma: 0.002; Simple hyperplasia versus carcinoma: 0.002; Complex hyperplasia versus carcinoma: 0.004 and *Chi-square test.

**Table 3:** Correlation between Wnt7a and ER/PR in endometrial carcinoma.

	Wnt7a versus ER		Wnt7a versus PR	
	*r*	*P*-value	*r*	*P*-value
Endometrial carcinoma	−0.281	0.019	0.249	0.037

*r*: Spearman's correlation coefficient; ER: estrogen receptor and PR: progesterone receptor.

**Table 4:** Univariate survival analysis of PFS and OS in 70 patients with endometrial carcinoma.

Variable	Number	Estimated 5-year PFS (%)	P*-value	Estimated 5-year OS (%)	P*-value
Age (years)			0.198		0.881
≤60	22	68.2		77.3	
>60	48	82.4		80.9	
FIGO stage			<0.001		0.016
I + II	39	94.7		89.7	
III + IV	31	56.1		66.7	
Grade			0.007		0.016
G1	30	93.3		93.3	
G2 + G3	40	65.3		69.2	
Lymph node metastasis			0.011		0.010
Negative	49	85.0		87.8	
Positive	21	60.0		60.0	
Depth of myometrial invasion			0.005		0.051
≤1/2	31	92.9		90.3	
>1/2	39	65.6		71.1	
LVS involvement			<0.001		<0.001
Negative	44	92.9		95.5	
Positive	26	50.6		52.0	
Peritoneal cytology			<0.001		<0.001
Negative	53	92.2		92.5	
Positive	17	27.3		37.5	
Wnt7a			0.005		0.042
Negative	44	66.5		72.1	
Positive	26	96.0		92.3	

FIGO: International Federation of Gynecology and Obstertics; LVS: lymph vascular space; PFS: progression-free survival; OS: overall survival and *log-rank test.

**Table 5:** Multivariate survival analysis of PFS and OS in 70 patients with endometrial carcinoma.

	HR	95% CI	P*-value
Progression-free survival			
FIGO stage	5.571	0.983–31.567	0.052
Grade	2.493	0.213–29.171	0.467
Lymph node metastasis	0.700	0.190–2.578	0.592
Depth of myometrial invasion	1.766	0.286–10.902	0.540
LVS involvement	3.983	0.463–34.249	0.208
Peritoneal cytology	8.497	1.664–43.376	0.010
Wnt7a expression	0.193	0.019–1.955	0.164
Overall survival			
FIGO stage	1.734	0.469–6.407	0.409
Grade	0.839	0.080–8.844	0.884
Lymph node metastasis	0.846	0.238–3.006	0.797
LVS involvement	7.683	0.867–68.043	0.067
Peritoneal cytology	5.082	1.258–20.525	0.022
Wnt7a expression	0.737	0.116–4.696	0.747

FIGO: International Federation of Gynecology and Obstertics; LVS: lymph vascular space; PFS: progression-free survival; OS: overall survival; HR: hazard ratio; CI: confidence interval and *Cox regression test.
